# A Turner Syndrome Patient Carrying a Mosaic Distal X Chromosome Marker

**DOI:** 10.1155/2014/597314

**Published:** 2014-03-17

**Authors:** Roberto L. P. Mazzaschi, Juliet Taylor, Stephen P. Robertson, Donald R. Love, Alice M. George

**Affiliations:** ^1^Diagnostic Genetics, LabPlus, Auckland City Hospital, P.O. Box 110031, Auckland 1148, New Zealand; ^2^Genetic Health Service New Zealand-Northern Hub, Auckland City Hospital, Private Bag 92024, Auckland 1142, New Zealand; ^3^Department of Paediatrics and Child Health, Dunedin School of Medicine, University of Otago, P.O. Box 913, Dunedin 9054, New Zealand; ^4^School of Biological Sciences, University of Auckland, Private Bag 92019, Auckland 1142, New Zealand

## Abstract

A skin sample from a 17-year-old female was received for routine karyotyping with a set of clinical features including clonic seizures, cardiomyopathy, hepatic adenomas, and skeletal dysplasia. Conventional karyotyping revealed a mosaic Turner syndrome karyotype with a cell line containing a small marker of X chromosome origin. This was later confirmed on peripheral blood cultures by conventional G-banding, fluorescence *in situ* hybridisation and microarray analysis. Similar Turner mosaic marker chromosome cases have been previously reported in the literature, with a variable phenotype ranging from the mild “classic” Turner syndrome to anencephaly, agenesis of the corpus callosum, complex heart malformation, and syndactyly of the fingers and toes. This case report has a phenotype that is largely discordant with previously published cases as it lies at the severe end of the Turner variant phenotype scale. The observed cytogenetic abnormalities in this study may represent a coincidental finding, but we cannot exclude the possibility that the marker has a nonfunctioning X chromosome inactivation locus, leading to functional disomy of those genes carried by the marker.

## 1. Introduction

Turner syndrome (TS) presents with a characteristic mild phenotype with some degree of variability [1]. The majority of patients have short stature, are infertile, and do not develop secondary sexual characteristics. Less consistent abnormalities include webbed neck, renal malformations (>50%), and cardiac defects (10%), while intelligence is considered normal. Approximately three-quarters of TS females inherit their X chromosome maternally [2].

Turner syndrome mosaics are also well documented and can be subcategorised according to whether the second cell line contains a whole or part of a sex chromosome. Jacobs et al. (1997) showed that, of 84 Turner syndrome cases with a standard karyotype of 45,X, 16% were mosaic, with a second cell line containing a ring X chromosome (45,X/46,X,r(X)) [3]. The phenotypic variability of these mosaics is largely dependent on the size of the ring and the presence of a functioning XIST.

XIST is a* cis*-acting gene in the X-inactivation centre (XIC), located in band Xq13. As a general rule, when one X chromosome has an imbalance that does not involve an autosome, the XIC on the abnormal X chromosome is activated. This activation leads to nonrandom skewing of X chromosome inactivation, with the XIST transcript inactivating the abnormal chromosome. The phenotype of this group of patients is generally that of a mild Turner variant phenotype [2].

Marker or ring X chromosomes (r(X)) lacking a functional XIST confer functional disomy of the duplicated region, which is expected to lead to a more severe phenotype. With the exception of mental retardation/developmental delay, they also share little phenotypic concordance. Migeon et al. (2000) described two TS variant mosaic cases with large imbalances and intact XIST regions on their markers [4]. These patients presented with mental retardation and fall into the category of “tiny ring X syndrome.”

The mechanism for generating ring chromosomes is thought to be initiated by two chromosome breakage events occurring at either side of a centromere, followed by fusion of the two broken ends of the centromere-containing fragment [1]. This event is thought to occur at meiosis. Non-ring marker chromosomes require the additional step of telomere addition/formation at the broken ends. Ring chromosomes bring the added complication of “ring chromosome syndrome,” whereby concentric rings are generated at cell division, with the inevitable nondisjunction, leading to more than one copy being present in some cells (also referred to as dynamic mosaicism).

Here we report a case which, from a cytogenetic viewpoint, appears to represent a relatively straightforward example of a Turner syndrome variant mosaic karyotype, with a small marker chromosome of X chromosome origin. However, from a clinical perspective, this case study has a rather severe phenotype that does not fit with patients previously reported in the literature. The final karyotype was based on a combination of FISH analysis, as well as conventional (G-banded) and molecular (microarray) karyotyping.

## 2. Case Report

The proband presented at nine months of age with poor growth/failure to thrive (below the 3rd percentile for her weight, length, and head circumference) and global developmental delay. There followed a lengthy period of deterioration, with additional problems including type I diabetes (at 10 years of age), clonic seizures, cardiomyopathy, hepatic adenomas, and skeletal dysplasia. Prior to initial karyotyping, a DNA sample was sent for entire mitochondrial DNA genome sequencing, which did not identify any pathogenic mutations. The gene for Wolcott-Rallison syndrome (WRS),* EIF2AK3*, was also sequenced but returned a negative result.

At 22 years of age she was assessed as functioning at the level of a 5-year-old child. She had delayed secondary sexual characteristic development and now presents with partial ovarian failure and growth hormone deficiency. The medical history of the rest of the family provided no additional information other than the proband having a brother with Asperger syndrome.

Three long-term closed flask fibroblast explant cultures were set up according to the protocol adapted from Rooney (2001) [5] when the proband was 17 years of age. G-banded chromosome preparations (at a resolution of 400 bands per haploid set) were made following the Seabright protocol [6]. 20/35 (57%) cells had a 45,X karyotype with a single X chromosome while the remaining cells had 46 chromosomes, with a single X chromosome and an additional small marker chromosome (possibly a ring), of unknown origin. Additional fluorescence* in situ* hybridisation testing was carried out on interphase nuclei of subcultured fibroblasts using the (Vysis) Aneuscreen FISH probe set (cenX (DXZ1), cenY (DYZ3), 13q14 (RB1), cen18 (D18Z1), and 21q22.13-q22.2 (D21S259/D21S341/D21S342)). In the case of mosaicism studies, a larger number of interphase nuclei were scored (at least 200), compared to our standard analysis of a minimum of 50 nuclei per probe set. The result was consistent with a female karyotype with a single X chromosome (Turner syndrome) in most nuclei, with a low-level two-copy X centromere signal pattern seen in 8/214 nuclei. The apparent discordance between the G-banded karyotype and later FISH analysis may be due to random loss of the marker cell line through continued subculturing.

**Figure 1:**
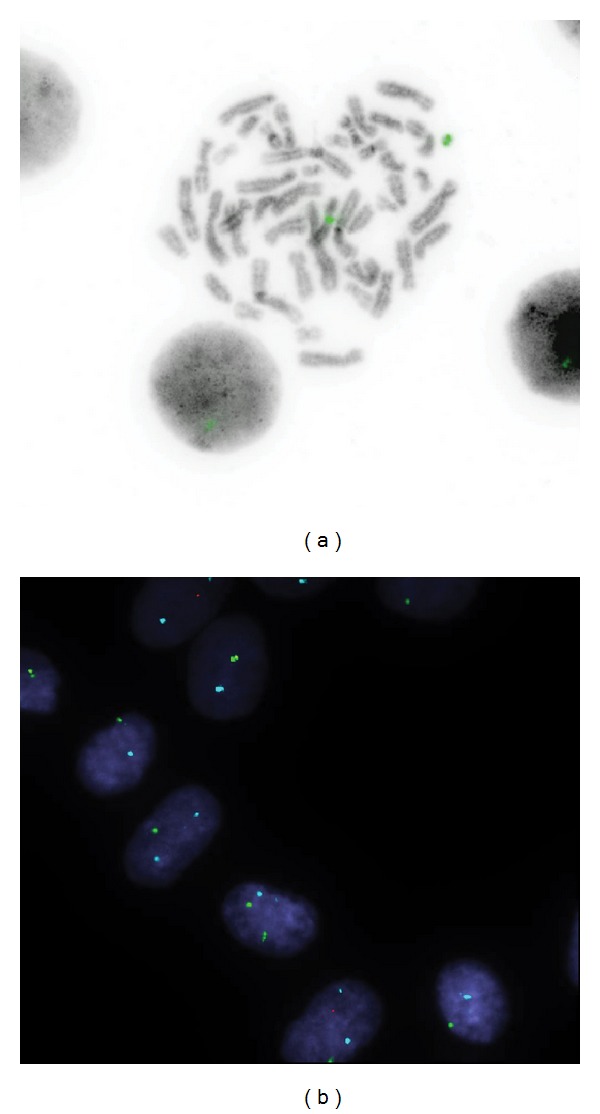
FISH analysis of the proband's cells. (a) The X chromosome centromere probe (Spectrum Green; DXZ1) on an inverted grey scale DAPI-stained metaphase spread shows hybridisation to both the normal X homologue and the marker chromosome. (b) X chromosome centromere probe (Spectrum Green; DXZ1) with a chromosome 18 centromere probe (Aqua; D18Z1) as a control. 1-2 X centromere signals per interphase nucleus can be seen.

A peripheral blood sample in lithium heparin was recommended at a higher chromosome band resolution (550 bands per haploid set) for further characterisation of the marker. G-banded (conventional) karyotype analysis on peripheral blood synchronised cultures using a modified method of Gallo et al. [7] confirmed the fibroblast culture findings, with 16/30 (53%) cells having a single X chromosome and 14/30 (47%) having the additional marker chromosome of unknown origin. FISH studies using the Vysis Xcen (DXZ1), Ycen (DYZ3), and Yp11.3 (SRY) probes on metaphase spreads were also carried out (Figure 1). The Xcen probe of centromeric alpha satellite DNA covered the region Xp11.1-Xq11.1 and hybridised to the normal X chromosome centromere and the marker in 9/30 (30%) cells. The remaining 21/30 (70%) cells, which lacked the marker, only showed hybridisation to the X chromosome centromere. This was considered to be a more reliable result than the previous interphase FISH of cultured fibroblast cells. The proportion of the two cell lines showed concordance with the G-banded karyotype results of both tissue types. No hybridisation of Y centromere or SRY probes were detected on the marker or other chromosomes. Parental blood samples were requested but never received.

**Figure 2:**
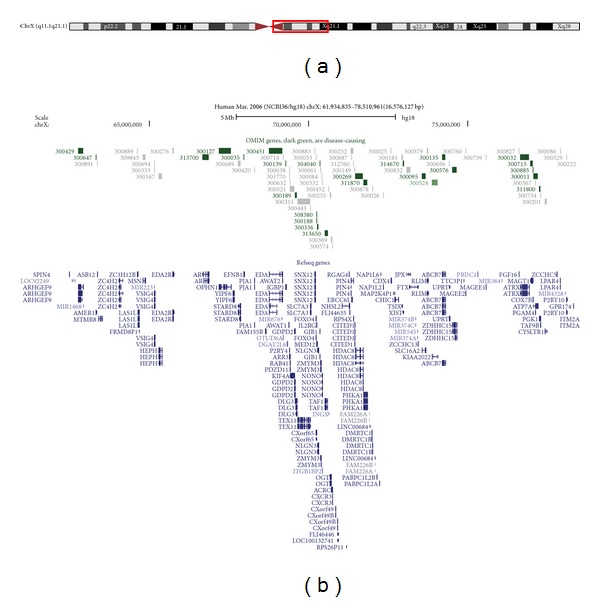
Schematic of the chromosome X region comprising the marker X chromosome (a) shows an ideogram of chromosome X, together with the region of the marker chromosome. (b) shows the OMIM and Refseq genes that lie on the marker chromosome. These graphics were taken from the UCSC genome browser (http://genome.ucsc.edu/).

Five years later an EDTA peripheral blood sample was received for molecular karyotyping as previously described [8]. Microarray analysis was carried out on extracted DNA using the Affymetrix Cytogenetics Whole Genome 2.7 M Array, Affymetrix Chromosome Analysis Suite (ChAS) v1.0.1/na30.1. An abnormal female mosaic molecular karyotype was determined as arr[hg18] Xq11.1q21.1(61,934,835-78,510,961)x1~2. This result indicated the presence of two genotypes: one with a single X chromosome complement, with no Y chromosome, and another of a single X chromosome complement with a 16.6 Mb duplication of X chromosome material from Xq11.1q21.1 (Figure 2), again with no Y chromosome.

An estimate of the level of mosaicism could not be made based on the microarray data. Taken together, the data suggest a mosaic marker chromosome comprised of an X centromere (from FISH) and pericentromeric euchromatin from the long arm of the X chromosome, including the X inactivation locus XIST. Due to the limits of the microarray assay (there is no probe coverage for centromeres or telomeres), the finding of X centromeric hybridisation to the marker detected on metaphase FISH could not be confirmed by molecular karyotyping.

Finally, molecular X chromosome inactivation analysis was attempted in order to determine if the marker chromosome was being expressed. If the marker was active, functional disomy for the included genes could provide an explanation for the observed severe phenotype. Unfortunately, X chromosome inactivation could not be assessed due to the lack of informativeness at the X chromosome amelogenin locus.

## 3. Discussion

In the case of a suspicion of Turner syndrome, a standard G-banded karyotype analysis is usually requested on a peripheral blood culture. Interestingly, the case reported here was not referred to confirm Turner syndrome but to confirm/exclude Wolcott-Rallison syndrome, which involved a number of metabolic and genetic tests.

Combining the conventional karyotyping and FISH data together with molecular karyotyping has allowed for the full characterisation of the genetic content of the marker chromosome. This marker contained many genes, including twenty-four classed by OMIM (Online Mendelian Inheritance in Man; http://www.ncbi.nlm.nih.gov/omim) as disease-causing. Of these,* OPHN1, IGBP1, DLG3, NLGN3, *and* ZDHHC15* are associated with mental retardation or Asperger syndrome phenotypes. Bedeschi et al. [9] described a case of a male with a duplication on the X chromosome from Xq12 to Xq13.1, a region that includes the* OPHN1* gene. He had severe mental retardation but an otherwise discordant phenotype compared to the case described here. This represents a much smaller duplication than our case study and was also nonmosaic but is another example of functional disomy of a part of the X chromosome. Hemmat et al. [10] described a rare case of an acentric marker X chromosome containing an activated neocentromere distal to this case study, at Xq21.2. This example raises the possibility of a similar sequence (of DNA with a degree of homology to centromeric sequences) being present in the marker of the case described here, which has been forced into activation to give it stability in cell division. This suggestion is compatible with the observed cytogenetic findings.

Migeon et al. [4] described two TS variant mosaic cases similar in appearance to our case study, but with larger imbalances, with intact XIST regions on their markers. They presented with mental retardation but an otherwise discordant severe phenotype to the case study reported here. These, as with most small r(X) cases in the literature, were published before microarray analysis was available, making gene content comparisons with this case difficult. It should also be noted that the marker chromosome is described as a ring based only on its G-banded appearance. However, telomere FISH studies have not been carried out, and so the possibility that the marker has telomeres, and is not a ring, cannot be excluded.

SNP (Single Nucleotide Polymorphism) analysis (data not shown) revealed homozygosity along the entire length of the X chromosome, including the region of disomy (the marker chromosome). Heterozygosity would have indicated the involvement of a second nonhomologous X chromosome (presumably from the other parent), in the formation of the marker. While not conclusive, our data suggest that the marker X may well have been derived from the normal X chromosome already present, rather than from a second chromosome X homologue.

Ring chromosomes also raise the possibility of “ring chromosome syndrome” occurring. At the DNA duplication phase of the cell cycle, concentric rings can accidentally be generated prior to cell division, with the inevitable nondisjunction leading to more than one copy of the ring segregating into some cells, which is also referred to as dynamic mosaicism. However, there was no evidence of “ring chromosome syndrome” in the G-banded analysis or FISH results. For non-ring marker chromosome formation, the additional steps of telomere generation and capping need to occur at the broken ends of the forming marker in order to confer chromosome stability.

For the purposes of this discussion, the mosaic marker can be considered as a 16.6 Mb gain of X chromosome material (from Xq11.1q21.1), against a Turner syndrome genotype background. As already discussed, the phenotypic effect of genes on the marker would only apply if a nonfunctioning copy of XIST was present. The marker did contain many genes, including twenty-four classed by OMIM as disease-causing (Online Mendelian Inheritance in Man (OMIM); http://www.ncbi.nlm.nih.gov/omim). Of these,* OPHN1*, * IGBP1*,* DLG3*,* NLGN3,* and* ZDHHC15* are associated with mental retardation or Asperger syndrome phenotypes. Bedeschi et al. [9] described a case of a male with a duplication on the X chromosome from Xq12 to Xq13.1, a region including the* OPHN1* gene. He had severe mental retardation, but an otherwise discordant phenotype compared to the case study. This is included here as it represents an example of functional disomy for the region of the X chromosome under investigation (although the reported case was much smaller, nonmosaic, and in a male).

Turner syndrome variants include female individuals with partial deletions in the “p” and/or “q” arms of one X chromosome. Deletions of certain X chromosome regions/genes can lead to specific phenotypic features which are characteristic of “full” or “classic” Turner syndrome. Deletions of the* SHOX* gene, located in the PAR (pseudoautosomal region) at Xp22.33, are associated with short stature although this characteristic TS feature is not noted in the case study. Primary ovarian failure (POF) has been associated with deletions of the* FMR1* gene (POF1) at Xq26–q28 [11] and the* DIAPH2* gene (POF2A) at Xq21.33 [12]. Type I diabetes has also been linked to the Turner syndrome phenotype [13].

The difficulty presented to the genetic counselors in this case was in trying to correlate the cytogenetic findings with the patient's phenotype. This patient was thought to represent an example of the severe end of the Turner syndrome spectrum, in tandem with poorly controlled diabetes. Hepatic adenomas had been previously reported in a child with Turner syndrome on growth hormone supplementation [14]. The karyotypic findings were considered to provide a unifying diagnosis for the patient's multiple comorbidities. The parents of this case study have not been karyotyped, making it impossible to give a risk of recurrence for future pregnancies. Similarly, the presence of this ring in a parent could also provide more information concerning a genotype-phenotype correlation. Transmission of ring X chromosomes has been previously described in both male and female offspring. However, although all these cases involved nonsupernumerary chromosomes, the rings were substantial in size with breakpoints more distal than those seen in this case [1].

## 4. Conclusions

This case study represents the coincidental finding of an individual with a severe set of clinical abnormalities and a Turner syndrome mosaic karyotype. The cytogenetic findings can be used to account for some of the observed phenotypic features, but the paucity of similar cases published in the literature makes a genotype-phenotype correlation difficult. We consider it likely that our patient's observed severe phenotype is due to functional disomy for those genes carried on the marker chromosome.
